# Monomelic, Multicentric, Metachronous Primary Chondrosarcoma of the Lower Limb: A Case Report

**DOI:** 10.7759/cureus.91869

**Published:** 2025-09-08

**Authors:** Prabodh Kantiwal, Divya Aggarwal, Siddhi Chawla

**Affiliations:** 1 Orthopedics, All India Institute of Medical Sciences, Jodhpur, Jodhpur, IND; 2 Pathology, All India Institute of Medical Sciences, Jodhpur, Jodhpur, IND; 3 Trauma and Emergency (Radiology), All India Institute of Medical Sciences, Jodhpur, Jodhpur, IND

**Keywords:** chondro-osseous tumor, endoprosthetic reconstruction, metachronous malignancy, multicentric chondrosarcoma, primary chondrosarcoma

## Abstract

An elderly man presented with a two-month history of swelling in the left lower leg. He had a prior history of chondrosarcoma of the first metatarsal, for which he underwent resection of the metatarsal and great toe in 2018. Current radiographs revealed an aggressive lytic lesion with mild sclerosis and cortical breach involving the medial tibial condyle. MRI demonstrated a heterogeneous lesion in the proximal tibia with multifocal cortical breaches and adjacent soft tissue involvement. Histopathological examination confirmed a diagnosis of low-grade chondrosarcoma. The patient subsequently underwent extended resection with endoprosthetic reconstruction.

## Introduction

Primary malignant bone sarcomas are exceedingly rare, representing only 0.2% of adult cancers. However, they span a diverse mesenchymal spectrum dictated by patient age, histology, cell of origin, intraosseous location, and pre‑existing skeletal condition [1,2]. Within the appendicular skeleton, osteosarcoma accounts for approximately 35% of cases, followed by chondrosarcoma (~30%) and Ewing sarcoma (~16%) [1]. The incidence of each tumor is strongly age‑dependent; Ewing sarcoma peaks in childhood, osteosarcoma in adolescence and young adults, and chondrosarcoma in later adulthood [3]. Chondrosarcoma is a malignant cartilage-forming tumor that arises either de novo, i.e., as a primary tumor, or from benign cartilage precursors such as enchondromas or osteochondromas, i.e., secondary tumors [4]. Conventional chondrosarcomas are graded I-III by cellularity and mitotic rate, while periosteal, clear‑cell, mesenchymal, and undifferentiated variants display distinct biology; low‑grade secondary lesions are typically indolent, whereas primary conventional and especially mesenchymal tumors are aggressive and prone to multicentricity and early metastasis [4]. Although ionizing radiation is an occasional culprit [5], most risk factors remain elusive; recent work implicates dysregulated Sonic‑Hedgehog signaling in malignant transformation and in the dense extracellular matrix, low proliferative index, and poor vascularity that render these tumors resistant to chemo‑ and radiotherapy [6]. Despite that resistance, conventional chondrosarcomas usually grow slowly and rarely metastasize, so surgical prognosis is excellent; recurrence or metastasis occurs in only a minority, and about 13% of recurrences “grade‑shift” upward [7].

Multicentric chondrosarcoma, characterized by multiple skeletal malignancies without visceral spread, is extraordinarily rare and can mimic osseous metastasis. It may present synchronously (as separate lesions) or metachronously (as new tumors appearing during follow-up after resection of an index lesion) [4,8]. Rigorous exclusion of overlooked baseline foci or local recurrence is essential before this diagnosis is made [9]. We describe just such a rarity: a metachronous, multicentric primary chondrosarcoma of the lower limb, with an initial Grade 2 tumor in the first metatarsal five years ago and a discrete Grade 2 lesion in the proximal tibia, an instructive reminder of the need for meticulous, long‑term skeletal surveillance in every chondrosarcoma survivor.

## Case presentation

A 71‑year‑old man presented with a painless, progressive swelling on the medial aspect of his left leg just below the knee, ongoing for two months. Five years earlier (2018), he had undergone resection of the left first metatarsal and great toe for a Grade 2 chondrosarcoma of the first metatarsal; margins were clear, and no metastases were detected, and he remained asymptomatic at that site.

**Figure 1:**
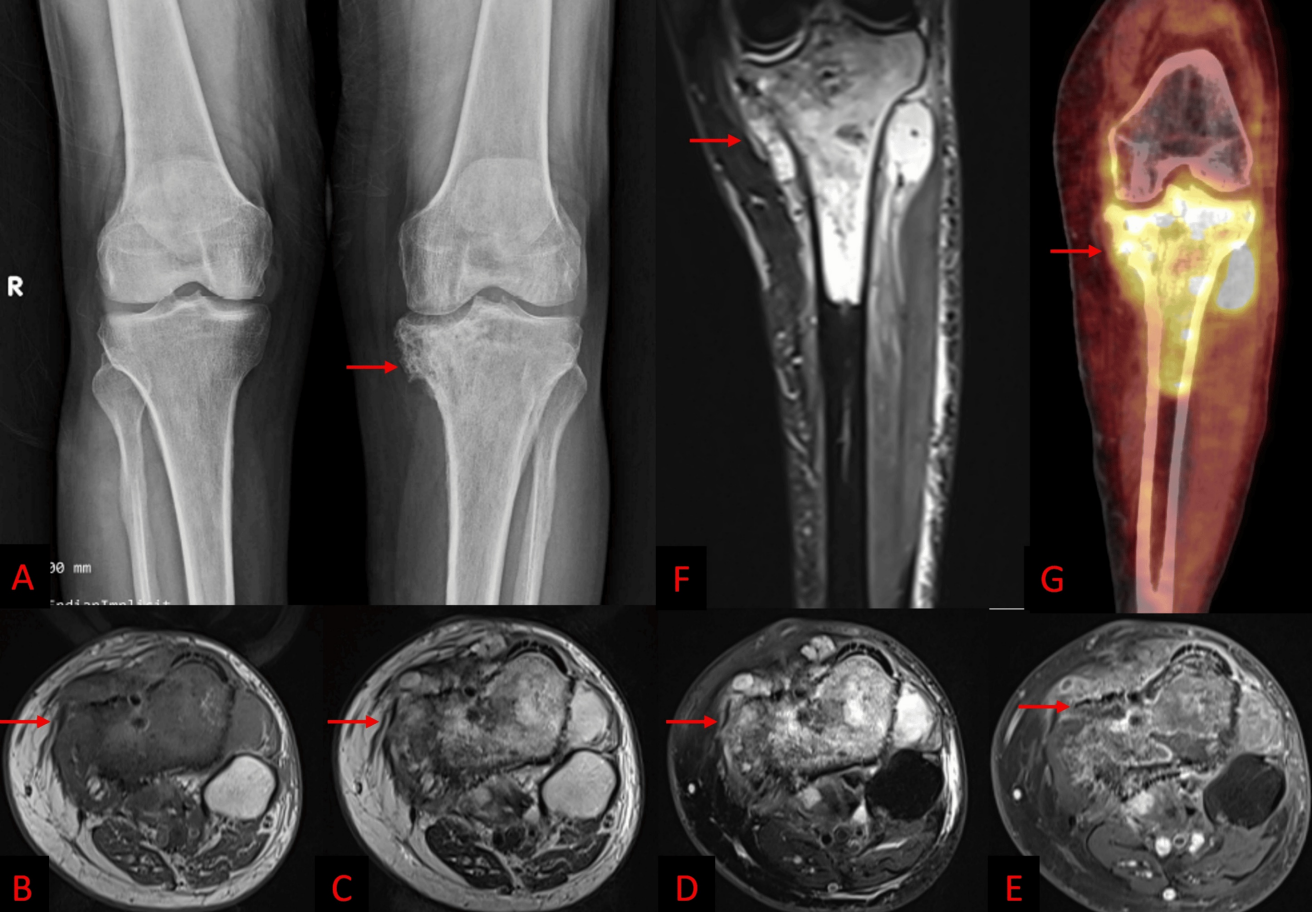
Anteroposterior radiograph of bilateral knees (A) shows a lytic lesion with irregular margins and sclerosis in the medial condyle of the tibia (red arrow). Axial T1-weighted (B), T2-weighted (C), fat-saturated T2-weighted (D), and post-contrast T1-weighted (E) MRI images show a heterogeneous lesion in the proximal tibia with enhancement and extraosseous soft tissue along the medial cortex (red arrows B-E). Coronal fat-saturated T2-weighted MRI (F) shows a well-defined lesion in the epiphyses and metaphysis of the tibia with extraosseous soft tissue (red arrow). Coronal PET-CT image (G) shows a hypermetabolic lesion in a similar region (red arrow). MRI: magnetic resonance imaging, PET-CT: positron emission tomography-computed tomography

Anteroposterior and lateral radiographs at presentation revealed a lytic‑sclerotic lesion involving the medial tibial condyle, marked cortical irregularity, and a wide transition zone. MRI confirmed a well‑defined epiphyseal-metaphyseal mass with multiple cortical breaches and an extra‑osseous soft‑tissue component, raising strong suspicion of recurrent chondrosarcoma (Figure 1).

**Figure 2:**
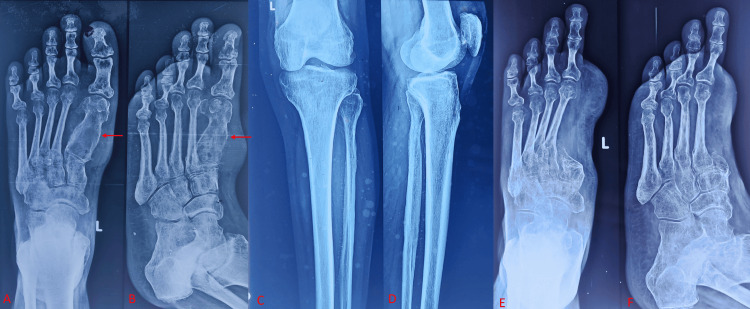
Previous radiographs from 2018 show (A) an AP view and (B) an oblique view of the foot, revealing a well-defined, lytic, expansile lesion in the first metatarsal with associated soft tissue (red arrows in A-B). (C) AP and (D) lateral radiograph of the knee with the leg is normal. Postoperative radiograph of foot (E) AP view and (F) oblique views, after excision of metatarsal and great toe. AP: anteroposterior

18F-FDG PET/CT demonstrated intense hypermetabolism confined to this tibial lesion, with no uptake at the prior metatarsal resection site and no evidence of distant disease (Figure 1). A retrospective review of his 2018 showed an expansile lytic lesion in the first metatarsal; however, radiographs showed no lesion in the tibia (Figure 2).

**Figure 3:**
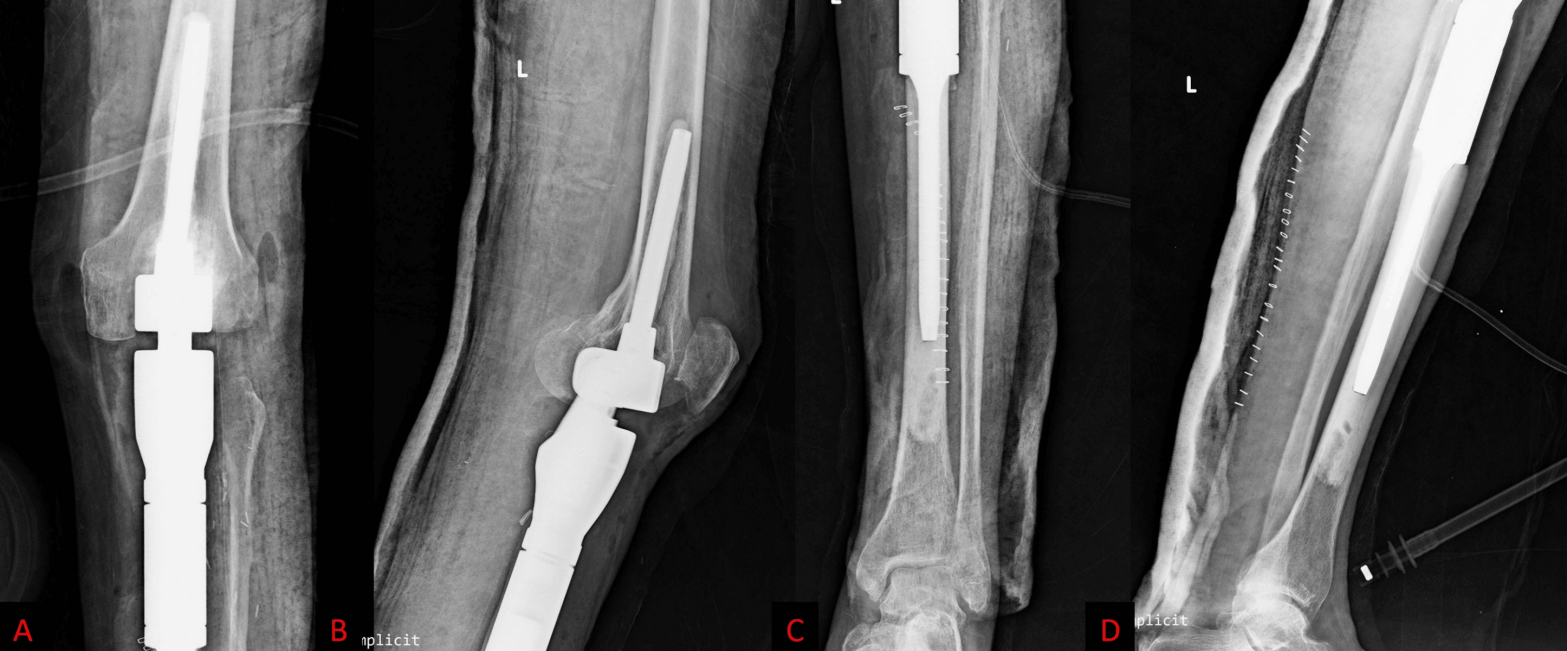
Postoperative X-ray: (A) AP view and (B) lateral view of the knee and (C) AP view and (D) lateral view of the leg reveal a metallic endoprosthesis in place after wide excision of the lesion in the proximal tibia. AP: anteroposterior

The patient underwent wide resection of the lesion in the proximal tibia with endoprosthetic reconstruction (Figure 3).

**Figure 4:**
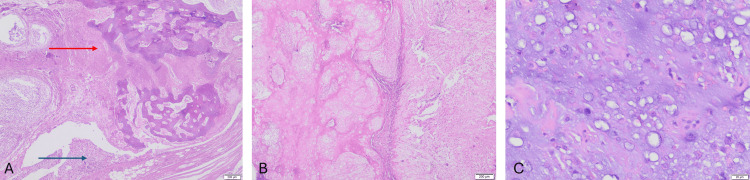
(A) Tumor with lobules of cartilaginous tissue permeating and entrapping bony trabeculae (red arrow) as well as infiltrating into adjacent soft tissue (blue arrow) (hematoxylin and eosin, 20x). (B) Lobules of cartilaginous tumor with increased cellularity (hematoxylin and eosin, 20x). (C) Tumor cells showing condensed nuclei with nuclear atypia and areas of increased cellularity (hematoxylin and eosin, 200x)

Histopathology revealed a lobulated, cellular cartilaginous tumor, separated by fibrous septae that contained congested vessels and entrapped bone trabeculae. Hyaline cartilage with increased cellularity and extensive myxoid change was noted; the tumor cells were small and ovoid, with mild to moderate nuclear atypia, hyperchromatic nuclei, inconspicuous nucleoli, and moderate eosinophilic to clear cytoplasm, accompanied by sparse mitotic figures, consistent with Grade 2 chondrosarcoma (Figure 4).

Postoperative recovery was uneventful, and he was discharged on day 7 with intact neurovascular status and partial-weight-bearing instructions.

## Discussion

Multicentric chondrosarcoma is extremely rare; the literature contains only scattered case reports, some confined to the skeleton and others extending to extra-skeletal sites [4,10-12]. In the largest single compilation to date, an eight-patient series published in 1996, five individuals presented with monomelic disease, three with disseminated lesions; three harbored synchronous tumors, five developed metachronous lesions, and one had underlying Ollier disease [12]. All synchronous cases were limited to a single limb, whereas every metachronous case, including ours, manifested its second tumor in a different bone.

Subsequent reports have primarily centered on cartilaginous dysplasia. Patients with Ollier disease or Maffucci syndrome often harbor multiple enchondromas detectable early on whole‑skeleton surveys, predisposing them to multifocal cartilaginous malignancy [13]. Our patient, however, displayed none of these dysplastic hallmarks, underscoring just how rare true multicentric, metachronous chondrosarcoma is in an otherwise normal skeleton.

Management of the patient was standard, and a multidisciplinary discussion was conducted. Finally, a decision was made to perform a wide local excision of the tumor with an endoprosthetic implant for the patient, citing the location in the epimetaphyseal region, the elderly age group's reduced need for ambulation, and the aim of avoiding future tumor recurrence. The limited data on previously published case reports [4,10-12] of monomelic multifocal chondrosarcoma have been managed according to patient-specific characteristics and the grade of the tumor on histology, with no clear-cut guidelines. All cases, however, advise routine postoperative follow-up of these patients for recurrence at the operative site or metachronous involvement of the other sites in the same limb. This case underscores the importance of vigilant, long‑term skeletal surveillance after resection of intermediate‑grade chondrosarcoma, as metachronous lesions may emerge years later and can be detected only through meticulous review of serial imaging.

## Conclusions

The case documents an exceptionally rare, metachronous presentation of multicentric Grade 2 chondrosarcoma in a patient without underlying cartilage dysplasia. Five years after curative resection of a first‑metatarsal lesion, a second, anatomically distinct tumor arose in the proximal tibia. The case highlights three key messages. First, true multicentric chondrosarcoma can develop in a seemingly normal skeleton and must therefore remain on the differential when new skeletal lesions appear years after the index surgery. Second, meticulous side‑by‑side comparison with baseline radiographs is critical; subtle, quiescent lesions may herald future disease and guide earlier intervention. Third, because most conventional chondrosarcomas respond poorly to chemotherapy and radiotherapy, timely wide excision remains the cornerstone of management, offering excellent functional and oncologic outcomes.
